# The Psychological Impact and Suicidal Behaviour in the Context of the COVID-19 Pandemic: Four Case Reports From Oman

**DOI:** 10.7759/cureus.50867

**Published:** 2023-12-20

**Authors:** Mandhar Al Maqbali, Ahmad Mohamed Eltanahy, Mohamed Elawdy, Salim AL-Huseini

**Affiliations:** 1 Psychiatry, Sohar Hospital, Ministry of Health, Muscat, OMN; 2 Anesthesia, Sohar Hospital, Ministry of Health, Muscat, OMN; 3 Urology, Sohar Hospital, Ministry of Health, Muscat, OMN; 4 Psychiatry, Al Masarra Hospital, Ministry of Health, Muscat, OMN

**Keywords:** coronavirus, oman, complex suicide, covid-19, covid

## Abstract

During the COVID-19 pandemic, quarantine has caused disruptions to daily social and economic activities. Many people have felt trapped and alone, experiencing rising levels of worry and financial hardships. Numerous studies have demonstrated that the COVID-19 pandemic increases depression and anxiety symptoms, as well as suicidal ideas and attempts, particularly in vulnerable individuals. We report four cases of suicidal attempts during the COVID-19 outbreak due to the lockdown and related financial difficulties. Those cases were admitted to a general hospital from April 2020 to June 2020. The patients were all male, had negative coronavirus tests, and committed violent suicides by hanging and slitting their throats. After receiving the appropriate treatment, all cases were discharged from the hospital. The COVID-19 pandemic and its economic and social impacts could result in significant consequences for vulnerable persons. Screening and early intervention play a role in averting the pandemic's mental health consequences.

## Introduction

The negative impact of the COVID-19 pandemic on public health is evident, with devastating morbidities and mortalities that were published in scientific journals. The adverse effects of the pandemic have extended to include a variety of psychological and mental illnesses [1,2]. Health quarantine, hospital admission in isolated units, personal equipment shields, and a daily negative news cycle all predispose to depression and increase the risk of suicide in COVID-19 patients, which have been reported recently [3,4]. Most governments enforced strict regulations and sanctions to limit the spread of the virus. This included face masks, social distancing, societal lockdown, and travel restrictions.

On the other hand, these measures increased the unemployment rate and threatened those working in small businesses and those who depend on a daily wage. All these factors affected the local and global economy with little cause for optimism [5,6]. Moreover, these factors not only compromise people who were affected by the virus but also COVID-negative cases at risk of suicide, with few published case reports [7,8]. We investigated a series of four expatriate workers who attempted suicide due to persistent feelings of isolation, financial troubles, and worries about their loved family members at home who were affected by COVID-19. All cases proved COVID-19-negative, all were saved and treated, and three were repatriated home at their request. Based on the premise that the risk of suicide is not only evident during the pandemic itself but also possibly in the months following its eradication [9], we aimed to raise the awareness of healthcare professionals about the detrimental psychological effects of COVID-19, as well as to draw attention to the individual psychological struggles and distinct elements that contributed to suicidal thoughts and actions in the Omani context during the pandemic. Additionally, it prompts the relevant national authorities to create and implement several plans that can help in averting similar unfortunate incidents. These can be accomplished in cooperation with charitable foundations to offer these disadvantaged communities financial assistance. The likelihood of suicide attempts could be reduced by combining all these strategies.

The preprint (version 2) of this article is available at Research Square (https://doi.org/10.21203/rs.3.rs-741841/v2).

## Case presentation

Between April and July 2020, we received information on four expatriate workers who attempted suicide and were successfully treated. All were male, had no significant medical diseases, and were healthy with no known psychiatric illnesses. The medical history taken from their friends and roommates referred to them as ordinary individuals with no bizarre behaviors. Although they have different jobs and careers, all shared similar social and financial circumstances: the economic shutdown, lost jobs, lack of alternative income, and three of them having severely affected COVID-19 family members in their home countries. Routine blood investigations were regular; urine toxicology screening was also negative.

Physical examination showed cut neck wounds in three and the hallmark of hanging in the fourth case. Patients with neck wounds were surgically treated, were admitted to the hospital for 10-14 days, and received psychotherapy. Three of them asked to travel to join their families, and after contacting their embassy, they were evacuated home, two in good general condition and one with tracheoesophageal fistula. The other demographics and data are in Table 1.

**Table 1:** Patients’ demographics, methods of suicide, reasons for suicide, management, and final disposal

Case	Personal, social history	Reason for attempting suicide	Method of suicide	Mental state exam (MSE)	Management	Disposal
Case (1)	26y, single, engineer	COVID-19 lockdown and border closure	Cutting his throat using a razor	He was cooperative, in a low mood with Reactive affect	Psychotherapy	was transferred to another hospital using medical evacuation
Case (2)	24y, single driver	Financial problems related to lockdown of COVID-19	Cutting his throat using a knife	Cooperative, Good eye contact, No delusional thinking or perceptual changes	Psychotherapy	Discharged
Case (3)	45y, married, restaurant worker	COVID-19 lockdown	Cutting his throat using a knife	Cooperative with euthymic mood and no delusional or perceptual changes	Psychotherapy	Evacuated to home country
Case (4)	37y, single, construction worker	COVID-19 lockdown	Hanging himself	Cooperative with euthymic mood and no delusional or perceptual changes	Psychotherapy	Evacuated to home country

## Discussion

Due to the COVID-19 societal lockdown, we introduced a case of four expatriate workers who felt isolated, had significant financial troubles, and could not travel home to support their families who were sick with COVID-19. They attempted suicide, all were saved, and three were evacuated home.

COVID-19 is an airborne virus that affects mainly the respiratory system and is transmitted primarily by droplet infection and direct contact. In response to the rapid spread of the virus, most governments have responded rapidly with social distancing, isolation, staying-home recommendations, and societal lockdowns to minimise the spread of the infection. Additionally, most governments had to close most of the international borders and delay the issuing of visas. Indeed, these measures helped minimise the pressure on the healthcare system, limiting the spread of the virus and lowering the rate of mortality that could be directly due to the virus. However, these sudden and repressive regulations are not free of negative consequences. They increase anxiety, stress, depression, and the risk of suicide [6,10]. The four cases in our series were typical examples of those negatively affected by the pandemic lockdown. They felt isolated and guilty as they could not travel to help their loved family members who were sick with COVID-19 and faced possible death. Social isolation contributes to the pathophysiology of psychiatric disorders and suicidal behaviour. Additionally, living alone gives rise to subjective feelings of depression, which are strongly associated with suicide, and that was reported in an extensive systematic review, which included 40 articles [11].

The lockdown affected the whole economy and mainly affected more non-governmental sectors, small businesses, the self-employed, and those drawing a daily wage. Despite the different jobs in our study, all had experienced major financial crises because of the lockdown and the subsequent effects on their daily income. The link between unemployment and economic troubles is known and has been reported in many published articles [12]. Although Hempstead et al. said that hanging is the most common method of suicide due to financial troubles, only one subject in our series attempted suicide by hanging, while the other three used knives [13]. This could be explained by the younger age range in our cases, and this is in agreement with Hempstead et al., who reported that the number of suicides using suffocation was 59.5% among those aged 40-64 years compared with 18.0% for those aged 15-39 years field [14].

The data obtained from the harmful effects of the 2008 World global economic crisis are an excellent example to justify the reported cases of suicide in Oman and other countries. In a study that included 54 countries, Chang et al. [14] said that 4,884 excess suicide cases in 2009 compared with the number expected based on previous trends (2000-2007) [15]. They concluded that, after the 2008 economic crisis, rates of suicide increased in European and American countries, respectively, particularly in men and in countries with higher levels of job losses. Similarly, the four cases in our series were male, and all lost their jobs.

Recent studies from different parts of the world on the COVID-19 pandemic showed similar findings. Initial reports from China by Wang et al. evaluated 1,210 people from other cities in China and found that 16% had symptoms indicative of moderate to severe depression [10]. In subsequent studies in the US conducted via Google Search, results that included the recent additions of words, phrases, and blogs that indicated depression and suicide, as well as phrases such as I lost my job, laid off, unemployment, and help, dramatically increased [6]. COVID-19 may have caused an increase in suicide risk factors that could yield long-term increases in suicidality and suicide rates [6].

A few case reports have been published in the US, the UK, Bangladesh, and India. Those cases presented findings based on the number of people who had committed suicide due to the combined factors of xenophobia, COVID-19, and negative media coverage [16]. The reports illustrated the use of different terminologies about the chosen method of suicide: gunshot [16], hanging [6], and knife [7]. In our series, hanging was used in one case, and the others used knives. It must be noted that it is challenging to access guns in Oman. Alleviating stress, anxiety, and feeling alone can be achieved by social media campaigns. Telephone calls, texts, and web chats can be used for this purpose, even in cases of societal lockdown. For example, RUOK (Are you OK?) is a non-profit suicide prevention charity launched in Australia Field [17]. It aimed to access people affected by COVID-19 and provide help to those at risk of suicide.

This research study provides a perspective on the psychological impact of the COVID-19 pandemic in Oman. It offers insights into cultural, societal, or individual factors influencing mental health outcomes, which enriches the broader understanding of the global impact of the pandemic on mental well-being. It was reported that the risk of suicide is not only evident during the pandemic, but also the rate may persist after its eradication [8]. The World Health Organization (WHO) announced that the pandemic may take a couple of years to control, and the complete eradication of the virus will not be achieved without developing an effective vaccine. The healthcare sector has to contact those patients who are known to have psychiatric diseases who are at risk for suicide. Additionally, the government and the media must encourage charity foundations to access the most badly affected and provide help and support. All those strategic measures could minimise suicide.

## Conclusions

Governmental measures, such as social distancing, lockdowns, and border closures, have slowed the spread of COVID-19. Still, they have also had psychological effects, such as a negative impact on finances, which has raised the chance of suicide. The present study offers valuable insights into the effects of the pandemic on mental health in Oman, particularly about suicidal tendencies. We suggest implementing online mental health intervention programmes to promote more reliable and authentic information about COVID-19 and make telemedicine care accessible. It is recommended that healthcare facilities, governments, and charitable foundations adopt preventive steps to avert such unfortunate incidents.
